# Targeting lipogenesis promotes the synergistic effect of the selective HDAC6 inhibitor ITF3756 with bortezomib in colon cancer cells

**DOI:** 10.3389/fphar.2025.1706770

**Published:** 2025-12-12

**Authors:** Marzia Franzò, Chiara Zichittella, Diana Di Liberto, Giovanni Pratelli, Federica Affranchi, Antonietta Notaro, Michela Giuliano, Sonia Emanuele

**Affiliations:** 1 Department of Biomedicine, Neuroscience and Advanced Diagnostics (Bi.N.D.), Biochemistry Building, University of Palermo, Palermo, Italy; 2 Department of Biological, Chemical and Pharmaceutical Sciences and Technologies (STEBICEF), University of Palermo, Palermo, Italy

**Keywords:** HDAC6 inhibitor (HDAC6i), bortezomib (BTZ), lipid metabolism, SREBP-1, apoptosis, colorectal cancer (CRC)

## Abstract

**Introduction:**

Selective histone deacetylase (HDAC) inhibition has recently emerged as a promising strategy for antitumor targeted therapy. HDAC6 is a member of the HDAC family that mainly deacetylates non-histone proteins, regulating multiple cellular functions, including lipogenesis. HDAC6 is associated with the development and progression of colorectal cancer (CRC) and is related to CRC poor prognosis. This paper evaluates the effects of the selective HDAC6 inhibitor ITF3756 in CRC cells in combination with bortezomib (BTZ), a proteasome inhibitor that promotes lipogenesis.

**Method:**

Cell viability was evaluated by MTT assay. Lipid content and quantification were estimated by ORO staining and triacylglycerol spectrophotometric kit. Apoptosis was detected by Annexin V/PI and cell cycle distribution analysis. Western blot was used to detect proteins involved in lipogenesis and apoptosis. SREBP-1 was knocked down by a specific siRNA.

**Results:**

The selective HDAC6 inhibitor ITF3756 reduced the viability of HCT116 and HT29 colon cancer cells and promoted lipogenesis. Considering the involvement of HDAC6 in controlling lipid metabolism, ITF3756 was combined with bortezomib (BTZ), a proteasome inhibitor that promotes lipid accumulation. Subtoxic doses of ITF3756 and BTZ exerted a synergistic apoptotic effect in HCT116 cells and caused mTOR phosphorylation, SREBP activation and PPARg increase, thus enhancing lipid production. The ITF3756/BTZ combination was less efficacious in HT29 cells that displayed a high basal level of lipid droplets. Diacylglycerol acyltransferase 1 (DGAT-1) and 2 (DGAT-2) inhibitors blocked lipogenesis and increased the effect of the ITF3756/BTZ combination in both cell lines, thereby suggesting that lipogenesis represents a defensive response. This hypothesis was confirmed by SREBP-1 silencing, which also potentiated the antitumor efficacy of the ITF3756/BTZ combination in HCT116 cells.

**Discussion:**

Overall, these results reveal a particular antitumor efficacy of the selective HDAC6 inhibitor in combination with BTZ in colon cancer cells and suggest that inhibiting lipogenesis is a useful tool to further increase the synergistic effectiveness.

## Introduction

Epigenetic targeting has been widely considered a promising tool in cancer therapy. Histone deacetylase inhibitors (HDACi) represent a class of epi-drugs that have revealed potent anti-tumor activity in different tumor models (Karati et al., 2024; Patel et al., 2023; Ramaiah et al., 2021). We have previously described the antitumor action of the pan-HDAC inhibitors SAHA (Vorinostat) and ITF2357 (Givinostat) in different tumor cells, outlining their action mechanisms (Carlisi et al., 2015; Celesia et al., 2022a; Zichittella et al., 2023; Celesia et al., 2023). Both compounds are ongoing in clinical trials for different types of tumors (Brown et al., 2021; Furlan et al., 2011; Galli et al., 2010; Godfrey et al., 2024; Pili et al., 2025; Rambaldi et al., 2010; Stitzlein et al., 2025) or already used in therapy. Specifically, Vorinostat is approved for the treatment of cutaneous T cell lymphoma and Givinostat for Duchenne muscular dystrophy (Borcoman et al., 2025). However, since Pan-HDACi have a broad-spectrum action towards histone deacetylases (HDACs), they may display a certain level of toxicity *in vivo* (Li et al., 2024).

Since specific HDAC family members, such as HDAC6, are overexpressed in different tumor types (Chen et al., 2022; Kim et al., 2022; Liu et al., 2016; Menbari et al., 2020), the development of selective HDACi has corroborated the idea of a focused targeted therapy, rather than using pan-HDACi that may exert multiple effects. Specifically, HDAC6, a member of the HDAC family exerting multiple functions, has been found increased in melanoma and colon cancers (Zhang et al., 2019; Vuletic et al., 2023; Hu et al., 2020). HDAC6 represents a unique HDAC in both structure and physiological functions (Pulya et al., 2021). Besides histone modification in the nucleus, HDAC6 localizes in the cytoplasm, where it targets several non-histone proteins including Hsp90, α-tubulin, HSF1 and others, modulating cellular functions (Vuletic et al., 2023; Pulya et al., 2021). It has been recently shown that selective HDAC6 inhibition suppresses tumor growth and restores sensitivity to chemotherapeutics (Oliveira-Silva et al., 2025a; Won et al., 2018) or antitumor agents (Huang et al., 2024; Peng et al., 2017).

In addition, an interesting link exists between HDAC6, and lipid metabolism in tumor cells (Qian et al., 2017; Zhong et al., 2025). Cancer cells can reprogram their lipid metabolism to sustain uncontrolled proliferation (Bacci et al., 2021; Yang et al., 2023). Recent evidence suggests that, beyond the well-known Warburg effect, certain cancer types can uptake and oxidize lipids highly sustaining mitochondrial oxidative phosphorylation in the presence of oxygen to produce a significant amount of ATP. Tumors may increase their lipid storage to easily mobilize these fuels to support cell growth and propagation (Cheng C. et al., 2018; Salita et al., 2022). Intriguingly, HDAC6 has been demonstrated to regulate fat-induced lipid storage (Qian et al., 2017) and to be inhibited by fatty acid supply in tumor cells (Ediriweera et al., 2021). More recently, Pant et al. provided evidence that the short-chain fatty acid butyrate potentiates the effects of HDAC6 inhibitors in cholangiocarcinoma cells (Pant et al., 2021). The involvement of HDAC6 in lipid metabolism is toward a decreasing effect on lipogenesis. Two different mechanisms in this regard have been characterised: 1) HDAC6 deacetylates CIDEC, a lipid storage mediator, thus reducing lipid accumulation (Qian et al., 2017). The same authors showed that fatty acid supply prevents HDAC6-mediated deacetylation of CIDEC, thereby favouring lipogenesis; 2) HDAC6 deacetylates FOXO, a transcription factor that promotes lipogenesis, when acetylated. Therefore, HDAC6 inhibition could promote lipogenesis (Lohner et al., 2024). ITF3756 is a novel HDAC6 inhibitor produced by Italfarmaco (Sandrone et al., 2021; Vergani et al., 2019). Recent evidence has been provided that ITF3756 has a substantial impact of on the chromatin landscape of cancer cells, including breast, leukaemia and melanoma (Zamperla et al., 2024). Moreover, HDAC6 inhibition by ITF3756 has been shown to modulate PD-L1 expression, thereby diminishing immune evasion and promoting T cell activation (Spadotto et al., 2025). Nevertheless, these represent the only published papers on the effects of this inhibitor in tumor cells and further studies are needed to investigate its anti-tumor effects.

It has long been known that HDAC6 is overexpressed in colon cancers (Zhang et al., 2019), therefore its inhibition may represent a targeted-based strategy (Vuletic et al., 2023).

Considering all these premises, the aim of this paper was to evaluate the effects of the selective HDAC6 inhibitor ITF3756 in CRC cells in combination with bortezomib (BTZ), a proteasome inhibitor that promotes lipogenesis (Xu et al., 2021). Specifically, the aim of this study was to investigate the antitumor mechanism of the ITF3756/BTZ combination and the impact of lipogenesis on tumor cell death using *in vitro* colon cancer models.

## Materials and Methods

### Cell culture

HCT116 and HT29 cells (ATCC-LGC Standards S.r.l., Italy) were cultured in Dulbecco’s Modified Eagle’s Medium (DMEM, Euroclone, United Kingdom) supplemented with 10% Fetal Bovine Serum (FBS), 1% Antibiotic Antimycotic Solution (100X, cat. No30-004-CI, Corning®, United States of America), 1% Sodium Pyruvate (100 mM, cat. No ECM0542D, Euroclone, United Kingdom) and 1% L-Glutamine 100X (200 mM, cat. NoAU-X0550-100, Aurogene S.r.l., Italy).

Cells were maintained in a humidified atmosphere 5% CO_2_ at 37 °C and used at early passages for all experiments.

### Chemicals and reagents

ITF3756 was synthesized and provided by the pharmaceutical company Italfarmaco S.p.A (Cinisello Balsamo, Milan, Italy), dissolved in DMSO (20 mM stock solution) and stored at −20 °C. For the experiments, the stock solution was diluted in DMEM, not exceeding 0.01% (v/v) DMSO, to realize the proper final concentrations.

Bortezomib (BTZ, Velcade or PS-341) was obtained from Millennium Pharmaceuticals (London, United Kingdom) solubilized in DMSO (5 mM stock solution), stored at −20 °C and used at different final concentrations.

The lipogenic inhibitors: DGAT-1i (Diacylglycerol Acyltransferase 1 A922500, cat. No 252801, Sigma-Aldrich, St. Louis, MO, United States of America) and DGAT-2i (Diacylglycerol Acyltransferase 2 PF-0642439, cat. No PZ0233, Sigma-Aldrich, St. Louis, MO, United States of America) were solubilized in DMSO or water according to the datasheet instructions (Stock solution: 5 mM and 10 mM, respectively), stored at −20 °C or 4 °C and used for the experiments at different final concentrations.

### MTT (3-[4,5-Dimethyl-2-thiazolyl]-2,5-diphenyl-2H-tetrazolium bromide) assay

Cell viability was evaluated by MTT assay (Cat. No M5655 6494, Sigma-Aldrich, United States of America) following the manufacturer’s instructions. Briefly, HCT116 and HT29 cells were seeded in 96-well plates (2.5 × 10^4^ cells/cm^2^) 24 h post-seeding, cells were treated with the compounds for the established time. MTT (1 mg/mL) was added for 2 h. The medium was then replaced with lysis buffer (20% sodium dodecyl sulfate, 40% N,N-dimethylformamide, pH 4.7) and the absorbance was measured by bio-photometer (Dynex Opsys MR™ Microplate Reader, Technologies, Chantilly, VA, United States of America) at 570 nm (Test wavelength) and 630 nm (Reference wavelength), using the lysis buffer as blank.

For synergism determination with combinations of ITF3756 and BTZ, HCT116 cells were treated with both compounds in a 3:5 ratio. The analysis and generation of the synergism curve were performed using CompuSyn Software version 1.0 (CompuSyn, Inc., Paramus, NJ, United States of America) and their synergy quantification using the Chou-Talalay method. The resulting combination index (CI) theorem of Chou-Talalay offers a quantitative definition for synergism (CI < 1), antagonism (CI > 1) and additive effect (CI = 1) in drug combinations (Chou, 2010).

In the experiments with lipogenesis inhibitors (DGAT-1i and DGAT-2i) and ITF3756-BTZ combination, HCT116 and HT29 cells were pre-treated for 1 h with the inhibitors (7.5 and 10 µM respectively). Then, ITF3756 and BTZ were added at the established concentrations for 24 or 48 h.

### Oil Red O staining

Oil Red O staining (ORO, cat. No O0625, Sigma-Aldrich, St. Louis, MO, United States of America) was used to detect neutral lipid content in HCT116 and HT29 cells, following the manufacturer’s instructions. ORO stock solution was prepared by dissolving 0.35 g of ORO in 100 mL of 100% isopropanol. Cells (2.4 × 10^4^ cells/cm^2^) were seeded in 24-well plates, allowed to adhere overnight and then treated with the compounds. After 48 h, the medium was removed and the cells were washed with Phosphate-Buffered Saline (PBS), fixed in 4% paraformaldehyde (PFA) for 10 min at room temperature, and washed with PBS. Then, 60% isopropanol was added for 15–20 s to remove PFA and then removed until completely dry.

Subsequently, a freshly prepared (3:2 ratio ORO stock solution diluted with distilled water) and filtered ORO working solution was added to the cells and incubated for 30 min at room temperature. ORO solution was then removed, and the cells were washed three times with distilled water. Stained cells were visualized under a Leica DM-IRB microscope at ×400 magnification. Representative images of the experimental conditions were acquired with the Leica DC300F digital camera. ImageJ software was used to quantify lipid droplets. First, the proper threshold was set and applied to all images, resulting in a binary image. Then, the pixels referred to the area covered by a single cell were used to measure the intensity of the lipid droplets in each image. The average of the intensity of an established number of areas was then normalized to the number of cells counted.

### Cell cycle distribution

HCT116 cells were seeded in six-well plates (1.8 × 10^4^ cells/cm^2^). 24 h post-seeding, cells were treated with 2 µM ITF3756, 5 nM BTZ either alone or in combination and maintained in a humidified atmosphere of 5% CO_2_ at 37 °C. After 48 h treatment, cells were harvested (0.025% Trypsin-EDTA), washed in PBS and resuspended in hypotonic solution (25 μg/mL Propidium Iodide, 0.1% Sodium citrate, 0.1% Nonidet P-40 and 10 μg/mL RNase A). The cell cycle phase distribution was evaluated by CytoFLEX LX Flow Cytometer (Beckman Coulter, Life Sciences, United States of America) using CytExpert 2.5 Software (Beckman Coulter, United States of America). Cell debris and aggregates were excluded by opportune gating, and 50.000 events were acquired for each sample.

### Annexin V/PI apoptosis detection assay

Annexin V/PI apoptosis detection assay (Annexin V-FITC Kit, cat. No 130-092-052, Miltenyi Biotec, Germany) was used to identify early and late apoptotic cells. HCT116 cells were seeded (1.8 × 10^4^ cells/cm^2^), allowed to adhere overnight, treated with 2 µM ITF3756, 5 nM BTZ either alone or in combination and maintained in a humidified atmosphere of 5% CO_2_ at 37 °C. After 48 h treatment, the percentage of cells in early or late apoptosis and cell necrosis was measured. Following the manufacturer’s instructions, cells were harvested, centrifuged and the resulting cell pellet was washed with PBS and resuspended in Annexin V binding buffer. The cells were then labelled with Annexin V and Propidium Iodide and incubated for 15 min at room temperature in the dark. Samples were then analysed by flow cytometry using a FACSCanto cytometer (Becton Dickinson, Franklin Lakes, NJ, United States of America). Approximately 50,000 events were acquired for each sample. Flow cytometry data were analyzed using FlowJo 10 software (BD Biosciences, San Diego, CA, United States of America), using a gating strategy excluding debris and doublet cells.

### Western blotting

HCT116 cells were seeded in six-well plates (1.8 × 10^4^ cells/cm^2^); 24 h post-seeding, cells were treated with 2 µM ITF3756, 5 nM BTZ either alone or in combination. At the end of treatment, cells were lysed using ice-cold lysis RIPA buffer (1% NP-40, 0.5% sodium deoxycholate, 0.1% SDS and protease inhibitors in PBS, pH 7.4) supplemented with a protease inhibitor cocktail for 20 min. Cell debris was removed by centrifugation and the supernatant, containing total protein lysate, was sonicated (10 s, three times at 10 rev) and quantified by Bradford assay (Pierce™ Coomassie Plus Assay Kit, cat. No 23236, Thermo Fisher Scientific, United States of America) using Bovine Serum Albumin as a standard (BSA, cat. No A2153, Sigma-Aldrich, United States of America), as previously reported (Zichittella et al., 2022).

The amount of 30 µg protein from each sample was separated by sodium dodecyl sulphate-polyacrylamide gel electrophoresis (SDS-PAGE) and transferred to nitrocellulose membrane. To verify the correct loading of all samples, the membranes were stained with 0.1% Ponceau Red in 5% acetic acid. The membranes were then washed with Tris-buffered saline-Tween 20 (TBS-T - 20 mM Tris, 140 mM NaCl, 0.1% Tween-20) and first incubated for 1 h in blocking solution (5% milk in TBS-T) at room temperature. After blocking, the filter was incubated overnight at 4 °C with primary antibodies: anti-p53 (DO-1, 1:200, cat. No sc-126, Santa Cruz Biotechnology, CA, United States of America), anti-caspase 3/anti-cleaved caspase-3 (1:1,000, cat. No 9662S, Cell Signaling Technology, United States of America), anti-PPAR-γ (1:1,000, cat. Nosc-7273, Santa Cruz Biotechnology, CA, United States of America), anti-SREBP-1 (1:1,000, cat. No#bs-140R, BioSS, Dundee, United Kingdom), anti-PARP-1 (1:700, cat. No sc-8007, Santa Cruz Biotechnology, CA, United States of America), anti-α-acetylated-tubulin (1:1,000, cat. No T6793, Sigma Aldrich, Milan, Italy) and anti-γ-tubulin (1:10,000, cat. No T6557, Sigma Aldrich, Milan, Italy), lamin-B (1:1,000, cat. No365962, Santa Cruz Biotechnology, CA, United States of America) and β-tubulin (1:1,000, cat. No sc-55529, Santa Cruz Biotechnology).

After washings in TBS-T, the membranes were incubated with appropriate HPR-conjugated secondary antibodies anti-Rabbit IgG (1:10,000, cat. No W4011, Promega Corporation, Madison, WI, United States of America) or anti-Mouse IgG (1:10,000, cat. No W4021, Promega Corporation, Madison, WI, United States of America) at room temperature for 1 h.

The chemiluminescent signal was visualized by chemiluminescence solution (ECL™ Prime Western blotting Detection Reagents, cat. No RPN2232, Amersham™, United Kingdom) and was detected using the ChemiDoc, XRS acquisition instrument (Bio-Rad, Hercules, CA, United States of America). The images were analysed using the Image Lab software (Bio-Rad, United States of America), using γ-tubulin as a housekeeping protein.

Depending on the molecular weight of the protein, if required, the membranes were subjected to stripping (15 g glycine, 1 g SDS and 10 mL TWEEN-20 in distilled water, pH 2.2), before proceeding with further incubation with other antibodies.

### Extraction of cytosolic and nuclear fractions

HCT116 cells were seeded in 60 mm-dishes (1.9 × 10^4^ cells/cm^2^); 24 h post-seeding, cells were treated with 2 µM ITF3756, 5 nM BTZ either alone or in combination and maintained in a humidified atmosphere of 5% CO_2_ at 37 °C. Cells were then washed in PBS and scraped in subcellular fractionation buffer (250 mM sucrose, 20 mM HEPES, 10 mM KCl, 1.5 mM MgCl_2_, 1 mM EDTA, 1 mM EGTA, 1 mM DTT, and protease inhibitors, pH 7.4). Subsequently, cells were passed 10 times through a needle of 25 G and kept on ice for 20 min. The homogenates were centrifuged at 720 g 4,700 rpm for 5 min at 4 °C. The pellets were resuspended in lysis buffer and passed 10 times through a 25 G needle and centrifuged again at 720 g 4,700 rpm for 10 min at 4 °C. The pellets (nuclear fraction) were lysed with nuclear buffer (standard lysis buffer with 10% glycerol and 0.1% SDS–1% NP-40, 0.5% sodium deoxycholate, 0.1% SDS, and protease inhibitors: s, 25 μg/mL aprotinin, 1 mM PMSF, 25 μg/mL leupeptin and 0.2 mM sodium pyrophosphate) and sonicated (10 s at 10 rev, three times). The supernatants obtained from the first centrifugation were considered as cytosolic fractions. Nuclear or cytosolic protein lysates were quantified by Bradford assay method and Western blotting was performed. Nuclear and cytosolic fractions were used to evaluate Cleaved SREBP-1. Lamin B and β-tubulin were used as nuclear and cytoplasmic protein housekeeping, respectively.

### Triacylglycerol quantification assay

The content of triacylglycerols (TGs) was quantified using a spectrophotometric commercial kit for TGs determination (Gesan Production s.r.l, Campobello di Mazara, Italy). HCT116 cells were seeded in 60 mm-dishes (1.9 × 10^4^ cells/cm^2^); 24 h post-seeding, cells were treated with 2 µM ITF3756, 5 nM BTZ either alone or in combination for 48 h. After removing the medium, cells were washed in PBS and lysed with 5% NP-40 on ice. Then, to solubilize TGs, the samples were repeatedly subjected to heat shock, slowly heated at 80 °C–100 °C and cooled to room temperature. After centrifugation, the reagent was added to the recovered supernatants and the samples were incubated for 10 min in the dark at room temperature. The amount of TGs for each sample was measured as absorbance using a Varian Cary 50 Scan UV-Visible Spectrophotometer at 546 nm and calculated by triacylglycerol standard curve (in nmol) and normalized to the same cell number (nmol/1 × 10^6^ cells).

### siRNA transfection

HCT116 cells were seeded in 96-well plates or in six-well plates at 2.5 × 10^4^ cells/cm^2^; 24 h post-seeding, cells were transfected with 80 pMoles/cm^2^ of Silencer® Select SREBP-1 (Cat. No #4390824, Thermo Fisher Scientific, United States of America), or Silencer® Select Negative Control (Cat. No #4390843, Thermo Fisher Scientific, United States of America). For cell transfection, Lipofectamine™ RNAiMAX Transfection Reagent (Cat. No13778030, Thermo Fisher Scientific, United States of America) was used following the manufacturer’s standard instructions. 16 h after transfection, the cells were treated with 2 µM ITF3756, 5 nM BTZ either alone or in combination in fresh medium and maintained for 24 h in a humidified atmosphere of 5% CO_2_ at 37 °C. Cells were then subjected to MTT assay or harvested to obtain protein lysates. The siRNA knockdown efficiency was assessed by Western blotting 40 h post-transfection.

### Statistical analysis

The data shown in all graphs represent the mean ± standard deviation (SD) of at least three independent biological replicates. Statistical analyses were conducted using the following tests: Student’s t-test (for comparing two groups), Ordinary one-way ANOVA (for comparisons across three or more groups) and two-way ANOVA (for the analysis of multiple variables between two groups). All analyses were performed using GraphPad Prism 10 software (GraphPad Software, United States of America).

P-values are indicated in the graphs as follows: * *p* < 0.05; ** *p* < 0.01; *** *p* < 0.001; **** p < 0.0001. p-value ≤ 0.05 was considered statistically significant.

## Results

### The effects of ITF3756 and bortezomib on colon cancer cell viability and lipid accumulation

**FIGURE 1:**
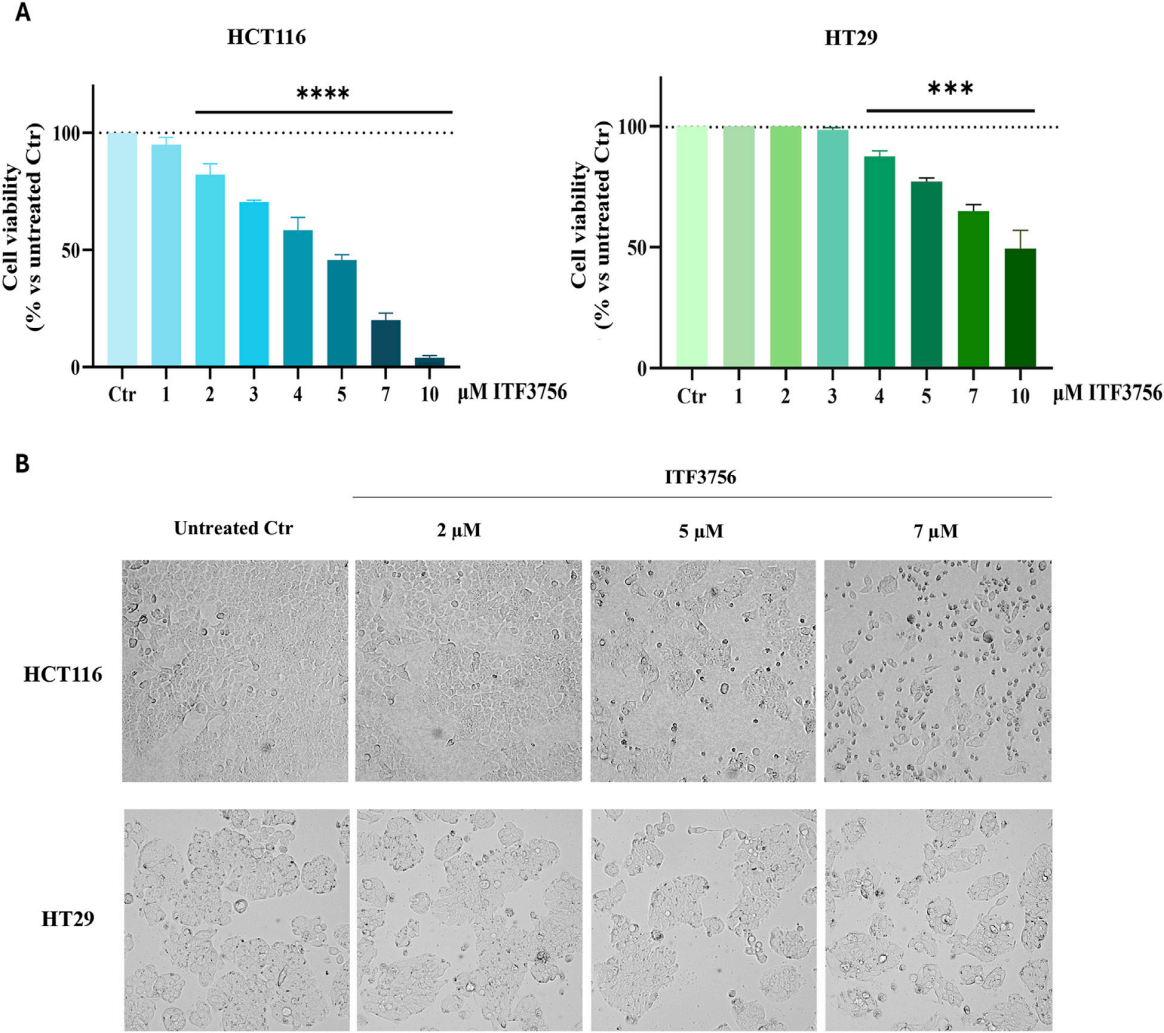
The Effects of ITF3756 on colon cancer cell viability and morphology. HCT116 and HT29 cells were treated for 48 h with increasing concentrations of ITF3756. Cell viability was evaluated by MTT assay. Data are expressed as cell viability percentages compared to untreated control (Ctr). The results reported in the graph are the mean ± SD of three independent experiments. Statistical analyses were performed using Ordinary one-way ANOVA with multiple comparison test, ***p < 0.001, ****p < 0.0001 **(A)**. The effect of different concentrations of ITF3756 on cell morphology **(B)** at 48 h treatment are shown. All images were visualized under a light microscope at ×200 (morphology) original magnification and acquired by OPTIKA PRO VIEW Digital Camera Software. The images are representative of three independent experiments.

Evidence has been provided in the literature that selective HDAC6 inhibition can induce lipid accumulation by different mechanisms (Qian et al., 2017). To test the efficacy of the selective HDAC6 inhibitor ITF3756 in colon cancer cells, we first evaluated its effects on cell viability and morphology in HCT116 and HT29 CRC cell lines. The results reported in Figure 1A show a comparative analysis indicating that the drug reduces the viability of both cell lines in a dose-dependent manner at 48 h treatment. The effects resulted more pronounced in HCT116 cells (about 50% cell viability reduction obtained with 5 μM), whereas this concentration only produced about 25% reduction in HT29 cells. Morphological analysis in HCT116 cells revealed that this concentration mainly produced a cytostatic effect and some morphological signs of cell death (cell shrinkage and detachment from the substrate), which were clearly visible and predominant with 7 μM ITF3756. On the other hand, HT29 appeared to be viable even with 7 μM ITF3756 but reduced in number (Figure 1B).

**FIGURE 2:**
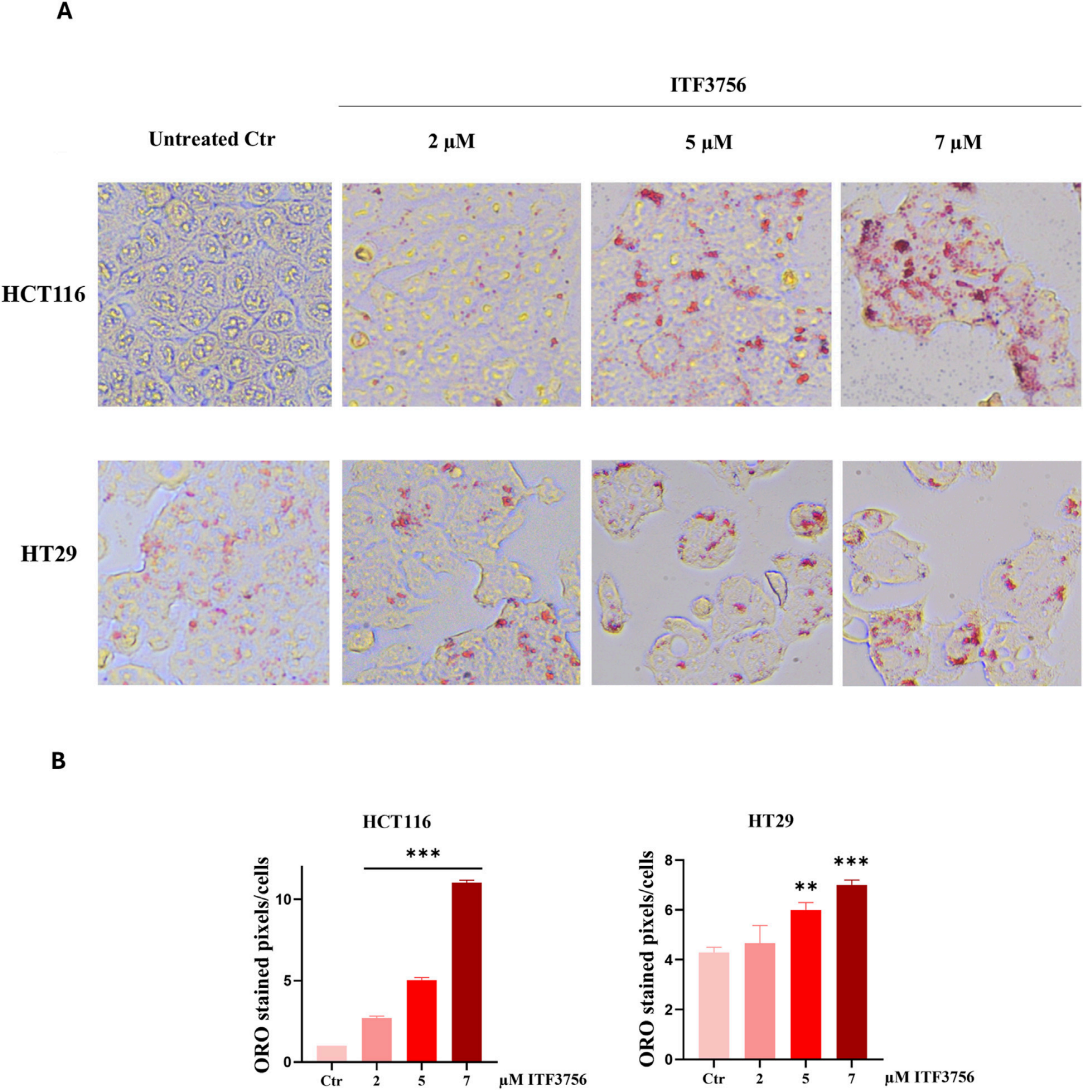
Lipid accumulation in colon cancer cells. The Effects of ITF3756. Representative Oil Red O staining images of HCT116 and HT29 cells treated with increasing concentrations of ITF3756 for 48 h **(A)**. The cells were visualized under a light microscope at ×400 magnification, and the pictures were acquired by OPTIKA PRO VIEW Digital Camera Software. Representative histograms showing quantification of ORO-stained image area in pixels corrected for cell number by ImageJ software **(B)**. Images and histograms are representative of three independent experiments. Statistical analyses were performed using Ordinary one-way ANOVA with multiple comparison test, **p < 0.01, ***p < 0.001.

Staining the cells with oil red O (ORO), a lipo-soluble dye, revealed that HT29 cells display a basal lipid droplet content which was not detected in HCT116 cells. As shown in Figure 2A, the HDAC6 inhibitor clearly produced lipid accumulation in HCT116 cells. This effect was evident at 5 μM and significantly increased with 7 μM ITF3756. Despite the high basal level of lipid droplets in HT29 cells, the HDAC6 inhibitor produced a dose-dependent increasing effect also in this case, being the lipid droplets bigger than those of the control and intensely stained. ImageJ quantification of lipid droplets confirmed these results as reported in the respective histograms (Figure 2B).

**FIGURE 3:**
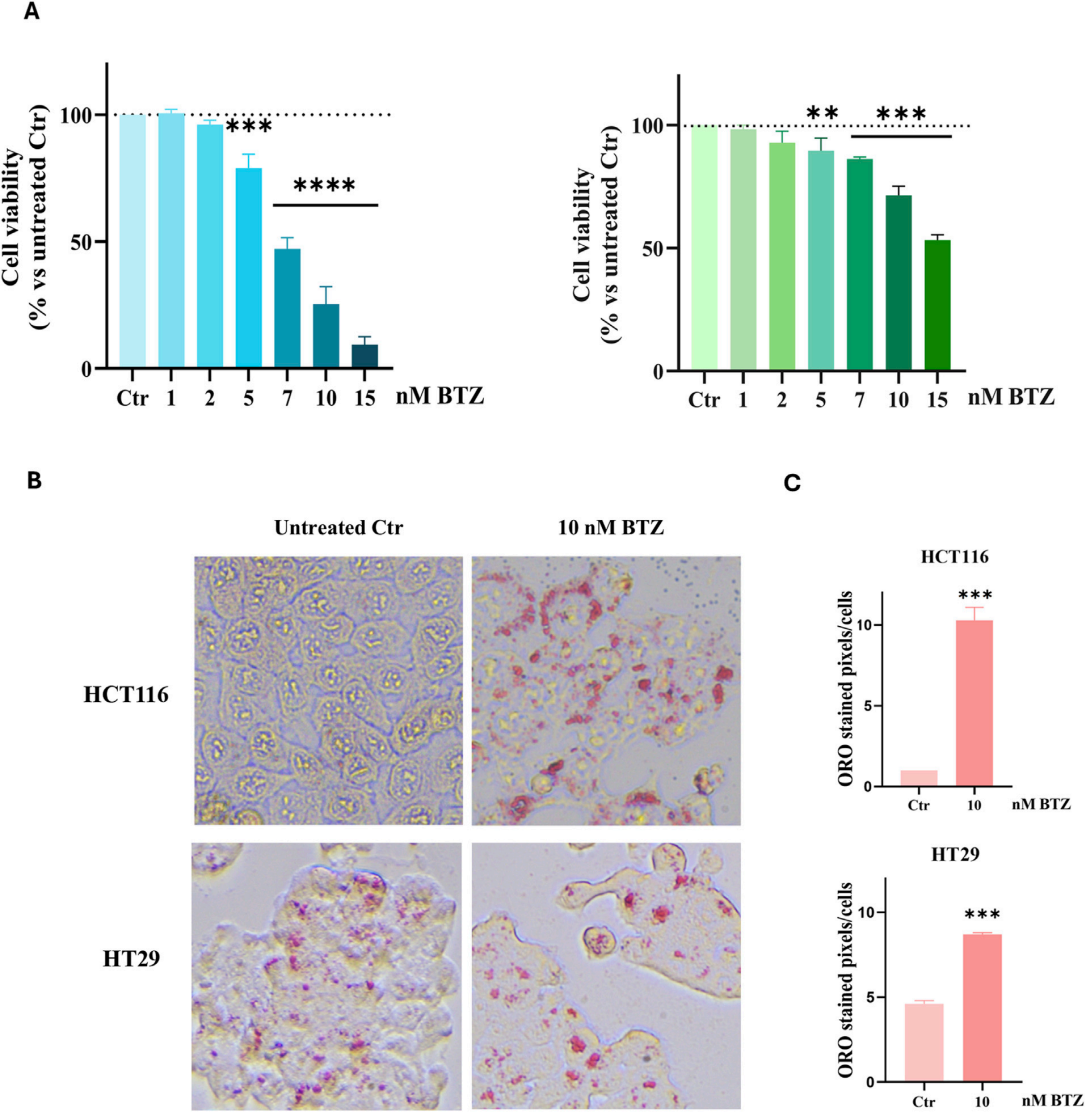
The Effects of bortezomib on colon cancer cell viability and lipid accumulation. Cell viability was evaluated by MTT assay in HCT116 and HT29 cells treated with different concentrations of bortezomib (BTZ), for 48 h **(A)**. Data are expressed as cell viability percentages compared to untreated control (Ctr). The results reported in the graph are the mean ± SD of three independent experiments. Statistical analyses were performed using Ordinary one-way ANOVA with multiple comparison test, ***p < 0.001, ****p < 0.0001. Representative Oil Red O staining images of HCT116 cells treated with BTZ for 48 h **(B)**. The cells were visualized under a light microscope at ×400 magnification, and the pictures were acquired by OPTIKA PRO VIEW Digital Camera Software. Representative histograms showing quantification of ORO-stained image area in pixels corrected for cell number by ImageJ software **(C)**. The images and the hystograms are representative of three independent experiments. Statistical analyses were performed using Ordinary one-way ANOVA with multiple comparison test, ***p < 0.001.

To clarify the role of lipogenesis and possibly induce a potentiation of the antitumor activity of the HDAC6 inhibitor, combinations with agents that also produce lipid accumulation were considered. Among the lipogenic compounds that we tested in our laboratory, the proteasome inhibitor bortezomib (BTZ) was selected for its ability to dose-dependently reduce the viability of both cell lines, even with a different efficacy (Figure 3A) and provoke dramatic lipid droplet formation at the active concentration of 10 nM (Figures 3B,C).

### The combination effects of ITF3756 and bortezomib

**FIGURE 4:**
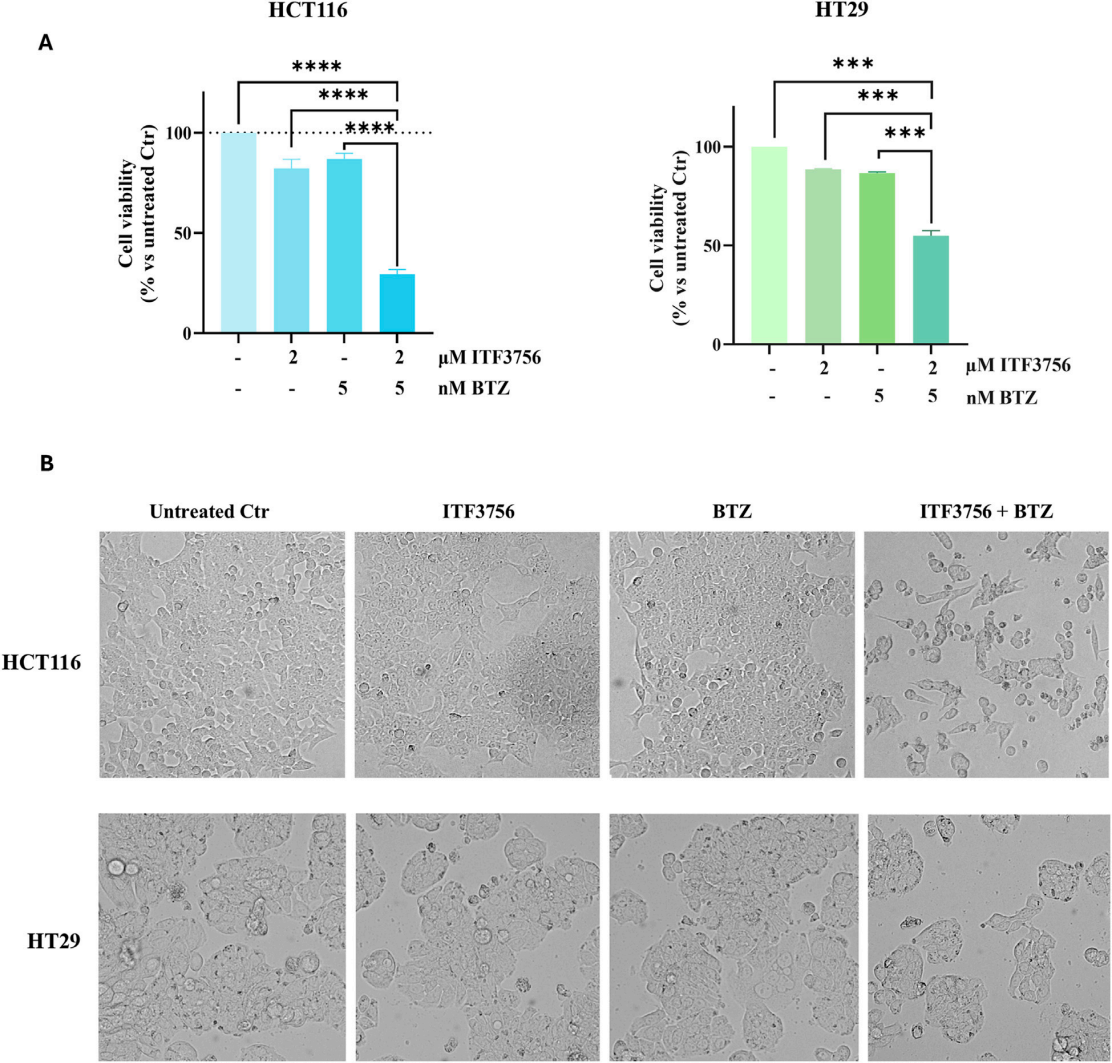
The combination effects of ITF3756 and bortezomib in colon cancer cells. HCT116 and HT29 cells were treated for 48 h with 2 µM ITF3756 and 5 nM BTZ either alone or in combination. Cell viability was determined by MTT assay. Data are expressed as cell viability percentages compared to untreated control (Ctr) **(A)**. Representative morphological images revealing the effects of the ITF3756/BTZ combination. Images were visualized under a light microscope at ×200 magnification, and the pictures were acquired by OPTIKA PRO VIEW Digital Camera Software **(B)** The results reported in the graphs are the mean ± SD of three independent experiments. Statistical analyses were performed using two-way ANOVA with multiple comparison test **(A)** and Student’s t-test **(B)**, ***p < 0.001, ****p < 0.0001.

For combination studies, as a starting point, 2 μM ITF3756 and 5 nM BTZ were chosen as subtoxic concentrations in both cell lines (Figures 1A, 3A). The histograms reported in Figure 4A show that associating the two compounds at these doses for 48 h produced a dramatic reduction of cell viability (about 70%) in HCT116 cells. Notably, the single compounds only slightly reduced cell viability, due to a modest cytostatic effect, as revealed by morphological analysis. In contrast, cells treated with the combination showed clear signs of cell death (Figure 4B). HT29 cells also responded to the ITF3756/BTZ combination, but the effects were less evident, being cell viability reduction about 50% (Figure 4A) with a lower extent of cell death and predominant reduction of the cell number (Figure 4B).

**FIGURE 5:**
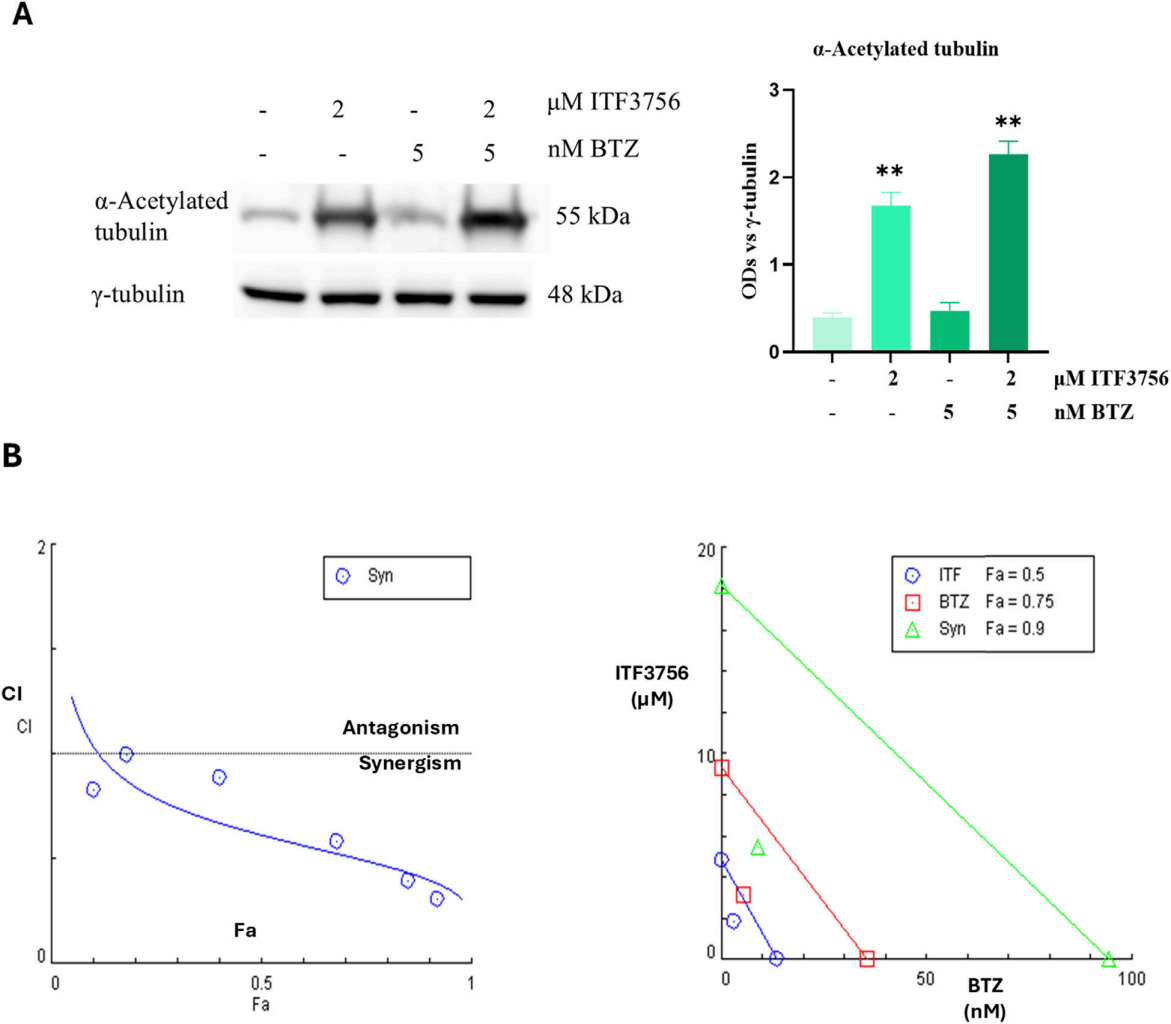
The selectivity of HDAC6 inhibition and the synergistic effects of the ITF3756/BTZ combination in HCT116 cells. Cells were treated for 48 h with 2 µM ITF3756 and 5 nM BTZ either alone or in combination. Representative images and densitometric analysis of western blots of Acetylated tubulin from HCT116 cells treated with ITF3756 and BTZ either alone or in combination at 48 h. The graphs show the OD (Optical Density) of the indicated proteins normalized for the housekeeping OD (γ-tubulin) **(A)**. Synergistic effects were evaluated after combining different doses of ITF3756 and BTZ, maintaining 3:5 ratio. The graphs represent the combination index plot (left) and the isobologram (right) obtained using CompuSyn software **(B)**.

Considering the higher susceptibility of HCT116, this cell line was mainly considered for subsequent experiments. First, to verify the selectivity of ITF3756 toward HDAC6, acetylated alpha-tubulin was evaluated. The results indicated that the compound, at 2 μM concentration, markedly increased alpha-tubulin acetylation in HCT116 cells either alone or in combination with BTZ (Figure 5A). Subsequently, to test whether the ITF3756/BTZ combination produces a synergistic effect, cell viability was evaluated at different doses in a 3:5 ratio. The results were then analysed using CompuSyn Software for synergism calculation developed by the mathematical model of Chou-Talalay. Data shown in Figure 5B confirmed that the selective HDAC6 inhibitor and BTZ exerted evident synergistic effect when used in combination.

**FIGURE 6:**
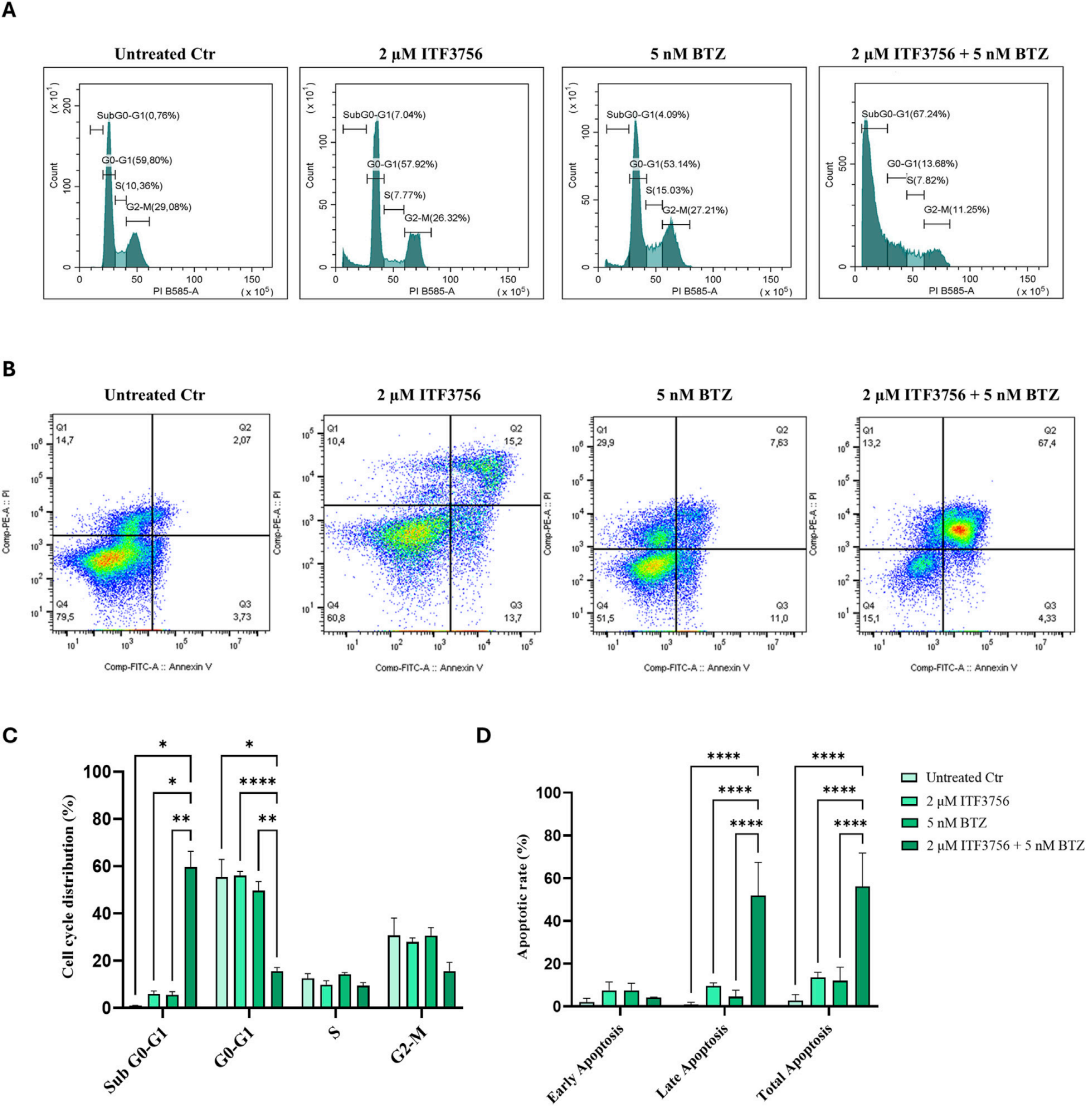
Flow cytometric analysis of cell cycle distribution and apoptosis induced by the ITF3756/bortezomib combination. HCT116 cells were treated for 48 h with 2 µM ITF3756 and 5 nM BTZ either alone or in combination. Hypotonic propidium iodide staining or propidium iodide/annexin V double staining were carried out to determine cell cycle distribution **(A)** or apoptosis **(B)**. The histograms indicate the percentage of cell cycle phase distribution **(C)** and the percentage of early apoptotic, late apoptotic and total apoptotic cells **(D)** compared to untreated control (Ctr). The results reported in the graphs are the mean ± SD of three independent biological replicates. Statistical analyses were performed using two-way ANOVA with multiple comparison test, *p < 0.05, **p < 0.01, ****p < 0.0001.

**FIGURE 7:**
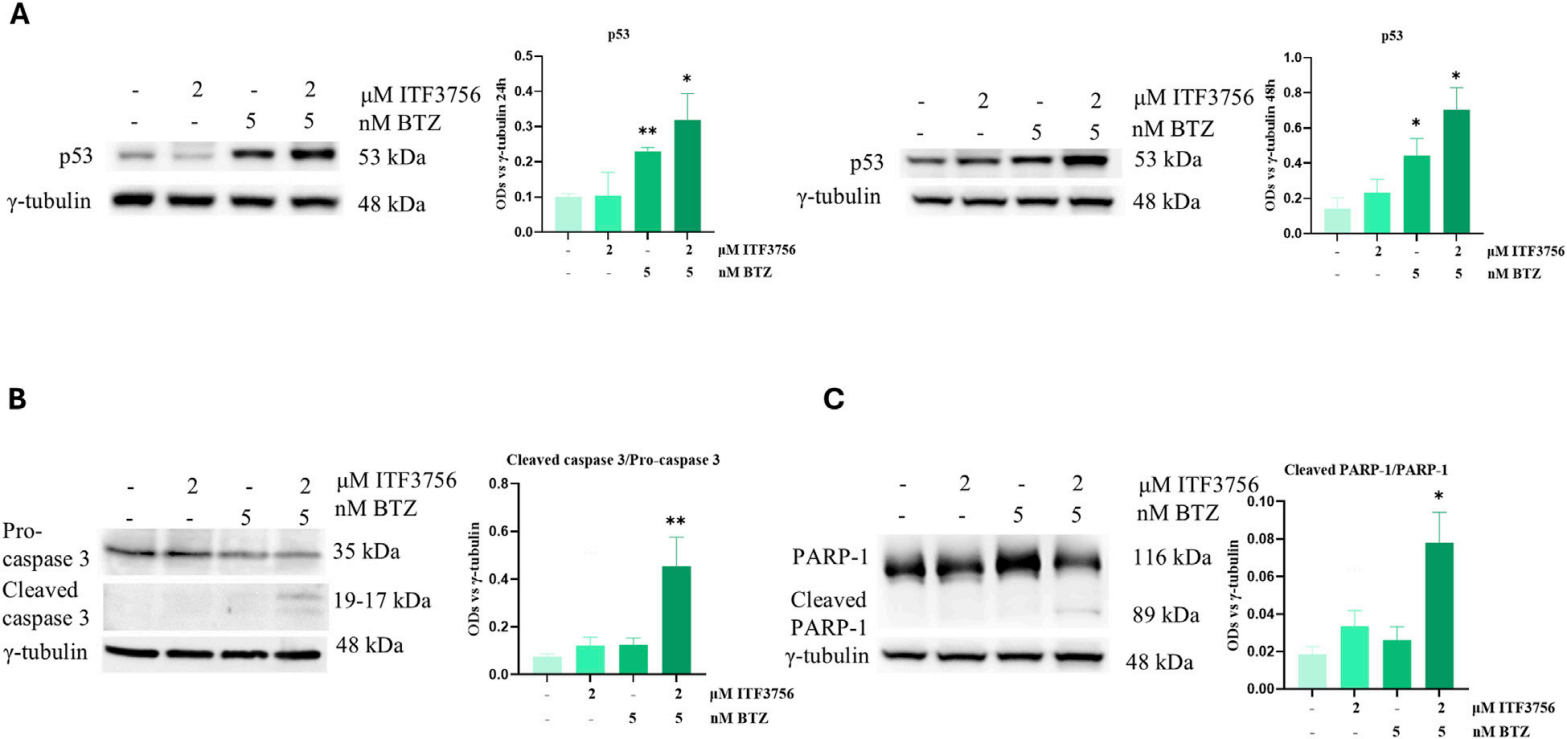
The effects of the ITF3756/bortezomib combination on apoptotic markers. Representative images and densitometric analysis of western blots of p53 **(A)**, pro-caspase 3 and cleaved caspase 3 **(B)**, PARP-1 and cleaved PARP-1 **(C)** from HCT116 cells treated with 2 µM ITF3756 and 5 nM BTZ either alone or in combination at 24 or 48 h (p53) or 48 h (caspase 3 and PARP-1). The graphs show the OD (Optical Density) of the indicated proteins normalized for the housekeeping OD (γ-tubulin). Data are expressed as the mean ± SD of three independent biological replicates. Statistical analyses were performed using Student’s t-test, *p < 0.05, **p < 0.01.

As confirmation, analysis of the cell cycle distribution by flow cytometry showed dramatic DNA fragmentation, detected by the subG0/G1 peak, which only occurred with the combination 2 μM ITF3756 and 5 nM BTZ at 48 h (Figures 6A,C). Moreover, apoptosis detection using annexin V and Propidium iodide (AV/PI) staining revealed a high percentage of double positivity only with the combination of the two compounds under the same treatment conditions (Figures 6B,D). Apoptosis induced by the ITF3756/BTZ combination was also shown to involve the tumor suppressor and pro-apoptotic protein TP53, as revealed by Western blot at 24 and 48 h (Figure 7A). Moreover, evaluating the protein levels of apoptotic markers at 48 h treatment revealed caspase 3 cleavage and PARP-1 degradation (Figures 7B,C), whereas these effects were totally absent with the single agents alone.

### The ITF3756/bortezomib combination produces lipid accumulation and SREBP-1 cleavage

**FIGURE 8:**
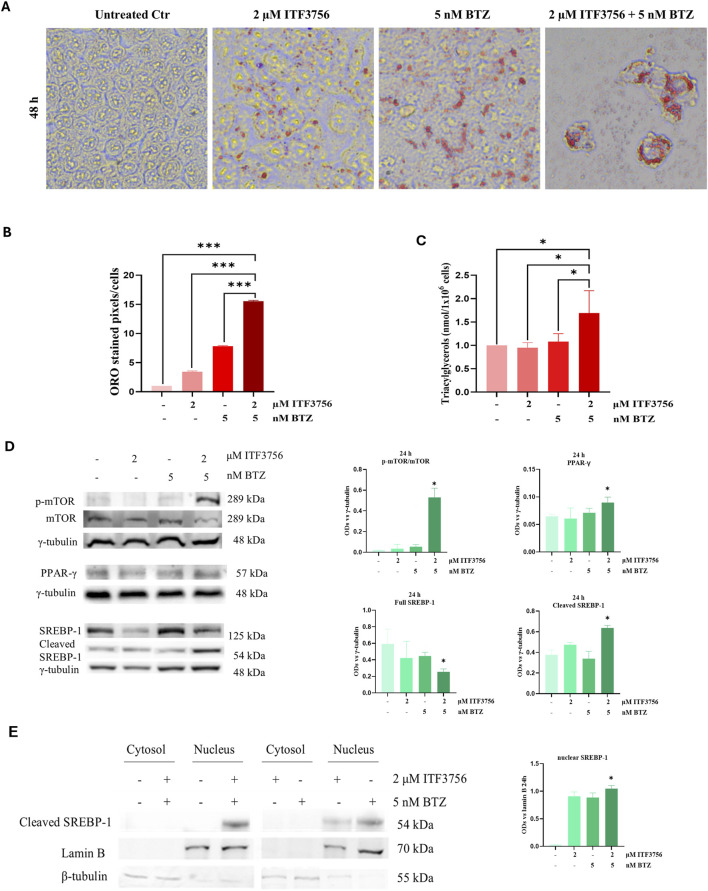
The ITF3756/bortezomib combination stimulates lipogenesis. HCT116 cells treated with 2 µM ITF3756, 5 nM bortezomib (BTZ) either alone or in combination for 48 h and subjected to ORO staining **(A, B)**, triacylglycerol quantification assay **(C)** or Western blot analysis **(D)**. For ORO staining, cells were visualized under a light microscope at ×400 magnification, and the pictures, representative of three independent experiments, were acquired by OPTIKA PRO VIEW Digital Camera Software. Representative histograms showing quantification of ORO-stained image area in pixels corrected for cell number by ImageJ software **(B)**. For triacylglycerol quantification, data are expressed as nmol/10^6^ compared to the untreated control (Ctr). Representative images and densitometric analysis of Western Blots of phospho-mTOR (p-mTOR), mTOR, full SREBP-1, cleaved SREBP-1 and PPAR-γ are shown. The graphs indicate the OD (Optical Density) of the proteins normalized for the housekeeping OD (γ-tubulin). All data are expressed as the mean ± SD of three independent biological replicates. Statistical analyses were performed using Student’s t-test, *p < 0.05. Cleaved SREBP-1 subcellular localization **(E)**. Representative images and densitometric analysis of western blots obtained from nuclear/cytoplasmic fractions of HCT116 cells treated for 24 h with ITF3756 and BTZ, either alone or in combination. The histogram shows the OD (Optical Density) of nuclear SREBP-1 normalized for nuclear housekeeping OD (Lamin B). Data are expressed as the mean ± SD of two independent experiments. Statistical analyses were performed using two-way ANOVA with multiple comparison test **(D)** and Student’s t-test **(D, E)**, *p < 0.05.

To investigate whether the ITF3756/BTZ combination produces lipid accumulation, ORO staining as well as triacylglycerol quantification assay were performed. The images reported in Figure 8A show that the single agents slightly provoked lipid droplets formation at 48 h treatment, while the effect was much more pronounced with the two compounds together. However, in this condition, the number of cells was reduced due to the action of the combination. Both lipid droplets quantification by ImageJ (Figure 8B) and triacylglycerols quantification (Figure 8C) confirmed these results. Indeed, the level of the lipids appeared significantly higher with the combination compared either with the untreated control or the single compounds.

Among the key players of lipogenesis, the mammalian target of rapamycin (mTOR), sterol regulating element binding protein 1 (SREBP-1) and peroxisome proliferator-activated receptor gamma (PPAR-γ) axis has been widely described (Cheng C. et al., 2018; Khan et al., 2024). Interestingly, Western blot analysis revealed dramatic phosphorylation of mTOR, which was detected in cells treated with the ITF3756/BTZ combination. This effect was accompanied by a decrease in the levels of the full-length SREBP-1, a lipogenic factor that is activated by proteolysis, as well as increase in the levels of the cleaved and active form (Cheng X. et al., 2018). In addition, a modest increase in the levels of PPAR-γ, another main promoter of triacylglycerol synthesis, was observed (Figure 8D). To confirm SREBP-1 activation, the translocation of the cleaved SREBP-1 active fragment was detected in the nucleus of HCT116 cells after treatment with the ITF3756/BTZ combination (Figure 8E). A faint band was also present after treatment with the single agents, thus confirming their role in promoting lipogenesis.

### Blocking lipogenesis increases the effects of the ITF3756/bortezomib combination

**FIGURE 9:**
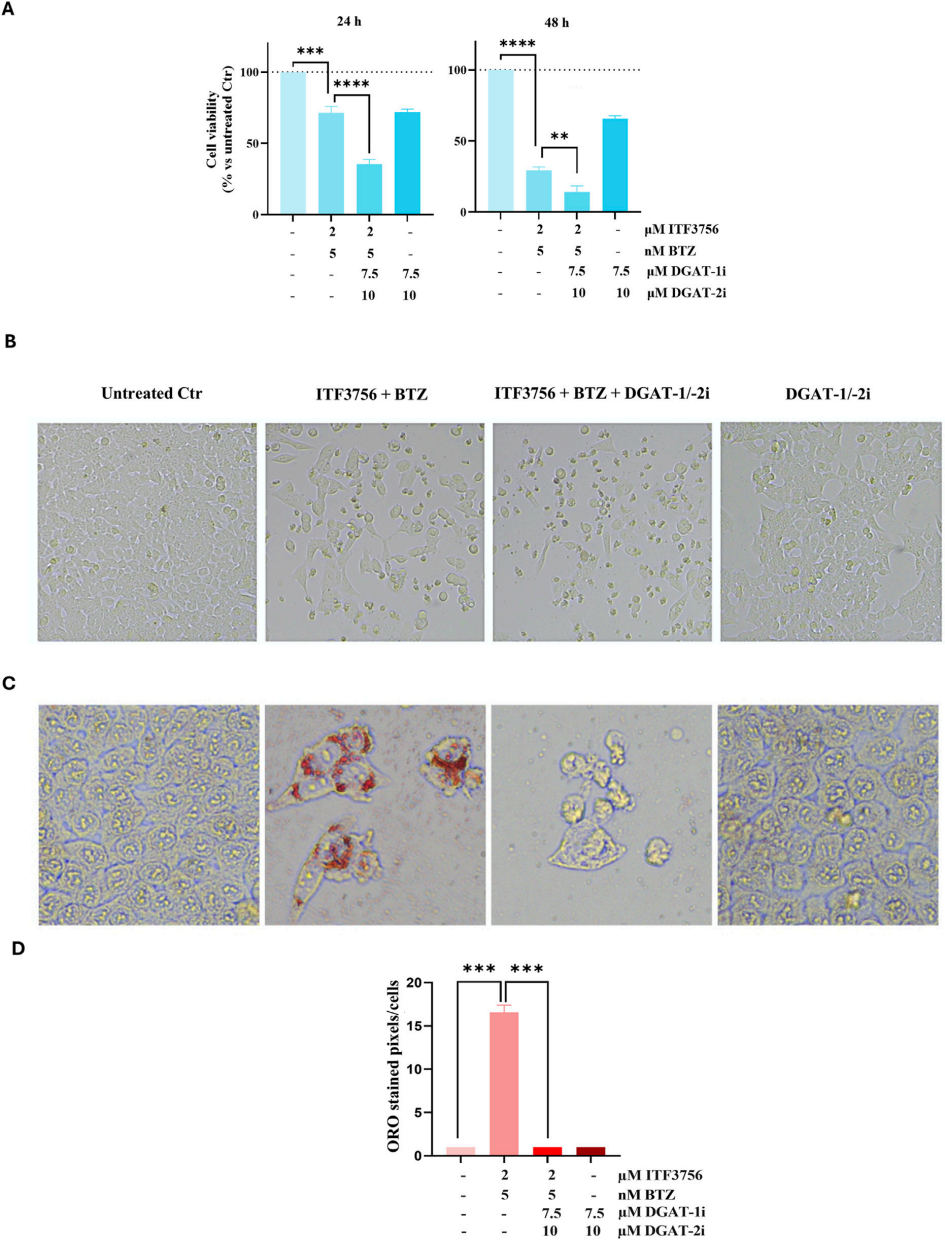
DGAT inhibitors prevent lipogenesis and exacerbate the effects of the ITF3756/bortezomib combination. Cells were pre-treated for 1 h with 7.5 µM DGAT-1 inhibitor and 10 µM DGAT-2 inhibitor. Then, 2 µM ITF3756 and 5 nM BTZ were added either alone or in combination for additional 24 or 48 h. Evaluation of cell viability **(A)** and cell morphology **(B)**. Treatment with single compounds in the presence of DGATi did not produce significant modifications (not shown). Data are expressed as cell viability percentages compared to untreated control (Ctr). Lipid accumulation was evaluated by Red Oil O staining **(C)** at 48 h treatment. All images were visualized under a light microscope at 200x **(B)** or ×400 **(C)** original magnification and acquired by OPTIKA PRO VIEW Digital Camera Software. The images are representative of three independent experiments. Representative histograms showing quantification of ORO-stained image area in pixels corrected for cell number by ImageJ software **(D)**. The results reported in the graph are the mean ± SD of three independent experiments. Statistical analyses were performed using two-way ANOVA with multiple comparison test, **p < 0.01, ***p < 0.001, ****p < 0.0001.

To clarify the role of lipogenesis induced by combination of the two compounds, lipogenesis inhibitors were used. Specifically, the diacylglycerol acyltransferases (DGATs), enzymes that promote triacylglycerol synthesis and consequent lipid droplets formation, were inhibited. To guarantee complete blockage of lipid droplets assembly, both DGAT-1 and DGAT-2 were inhibited with specific inhibitors. As described in Materials and Methods, concomitant pretreatment for 1 hour with DGATi was performed and then ITF3756 and BTZ were added for the established times and concentrations. The results shown in Figure 9 indicate that the effect of the ITF3756/BTZ combination increased in the presence of DGAT inhibitors (DGATi). This was observed either by cell viability evaluation (Figure 9A), or cell morphology observation (Figure 9B). Indeed, the viability of the cells treated with the ITF3756/BTZ combination in the presence of DGATi was further reduced, while the reduction observed with DGATi alone was attributable to a modest reduction in cell number, being the cells viable (Figure 9B). Notably, the cells that were pre-treated with DGATi and then subjected to the ITF3756/BTZ combination showed further signs of cell death compared to those treated with the two compounds in the absence of DGATi.

As a confirmation of the DAGTi efficacy, ORO staining permitted to verify that the two lipogenesis inhibitors completed prevented the effect of the combination ITF3756/BTZ on lipid droplets formation (Figures 9C,D).

**FIGURE 10:**
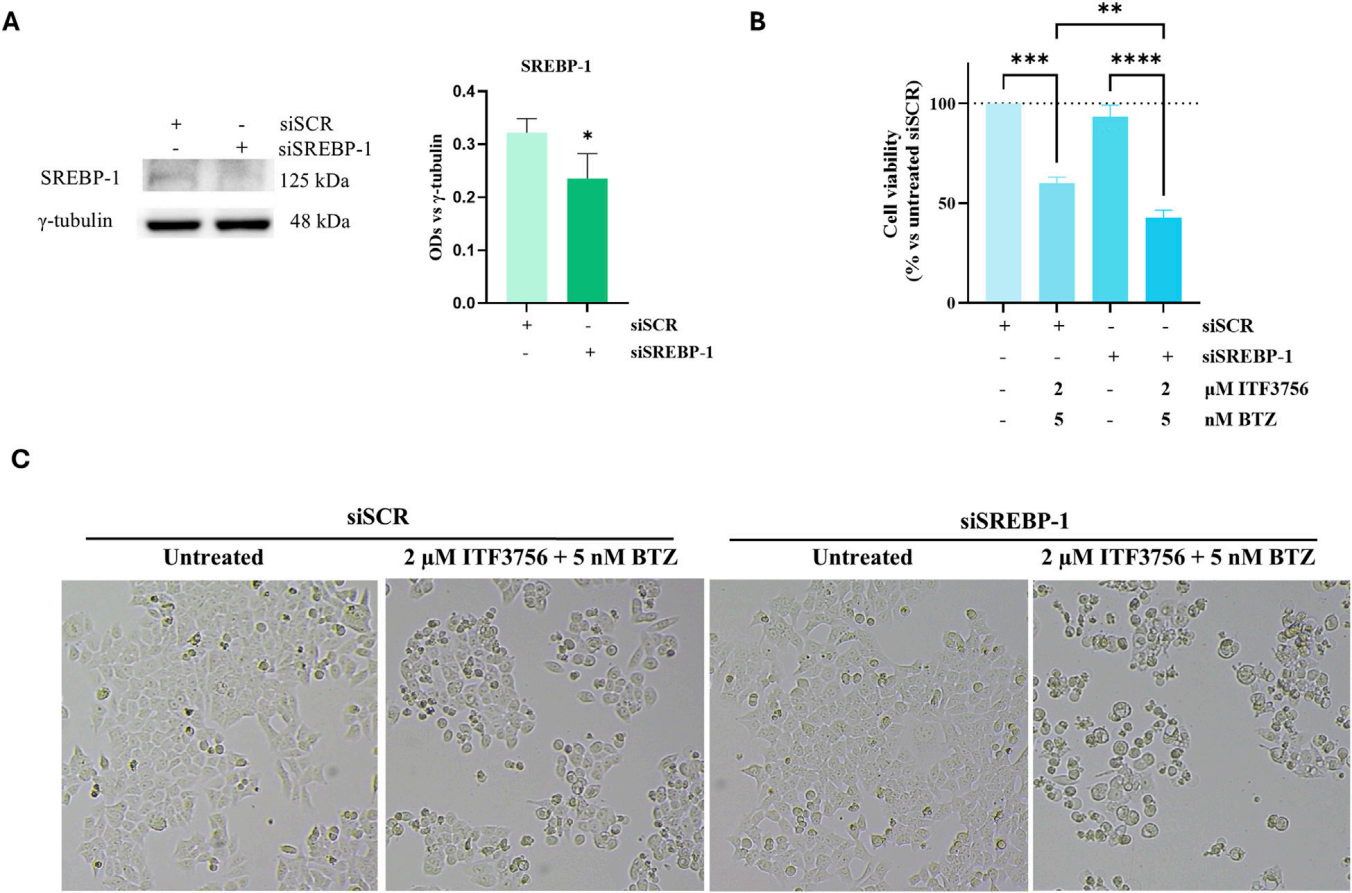
SREBP-1 silencing increased the effects of the ITF3756/bortezomib combination. Cells were transfected for 16 h with 80 pMoles/cm^2^ of Silencer® Select SREBP-1 (siSREBP-1), or Silencer® Select Negative Control (siSCR). The silencing efficiency was determined by SREBP-1 Western blot **(A)**. After transfection, cells were treated with 2 µM ITF3756 and 5 nM BTZ in combination for additional 24 h and subjected to cell viability **(B)** and cell morphology **(C)** evaluation. Data are expressed as cell viability percentages compared to untreated siSCR control. The results reported in the graphs are the mean ± SD of three independent experiments. Statistical analyses were performed using Student’s t-test **(A)** and two-way ANOVA with multiple comparison test **(B)**, *p < 0.05, **p < 0.01, ***p < 0.001, ****p < 0.0001. Images were visualized under a light microscope at ×200 magnification, and the pictures were acquired by OPTIKA PRO VIEW Digital Camera Software. The images are representative of three independent experiments.

Given the role of SREBP-1 in lipogenesis and our results indicating its activation and nuclear translocation, we decided to knockdown this factor by a specific SREBP-1 siRNA to corroborate the data obtained with DGATi. As shown in Figure 10A, SREBP-1 siRNA transfection produced a significant reduction in the SREBP-1 protein level. Moreover, the effect of the ITF3756/BTZ combination was more pronounced in SREBP-1 silenced cells compared to scramble control cells, as revealed by morphological analysis (Figure 10B) and evaluation of cell viability (Figure 10C).

To further verify the role of lipid droplets and validate the combination strategy, experiments to inhibit lipogenesis were also performed in HT29 cells. Data reported in Supplementary Figure S1 indicate that DGATi completely abolished lipid accumulation and exacerbated the effects of the ITF3756/BTZ combination, thereby confirming that colon cancer lipogenesis exerts a pro-survival role.

## Discussion

This paper provides evidence that the selective HDAC6 inhibitor ITF3756 exerts a synergistic interaction with the proteasome inhibitor bortezomib. Although others have previously shown that selective HDAC6 inhibition produces anti-tumor effects synergistically augmented by bortezomib, a precise mechanism has not been described. Most papers, indeed, ascribe this interaction to a stress condition that arises from proteasome inhibition and impaired aggresome formation, a cellular structure sequestering misfolded or non-degraded proteins. In particular, Huang et al. have shown that a combination of a HDAC6 inhibitor and bortezomib increases ubiquitinated proteins due to aggresomal pathway inhibition, thereby leading to apoptosis (Huang et al., 2019). Mechanistically, they propose that the HDAC6 inhibitor prevents the binding of HDAC6 with dynein, a motor cytoskeleton protein, thus blocking the transport of misfolded proteins to the aggresome.

It is not surprising that most of the studies describing the synergistic effect of HDAC6 inhibitors with bortezomib have been referred to multiple myeloma (MM) cells. As it is well known, bortezomib is the first FDA-approved proteasome inhibitor for treating multiple myeloma at different stages (Sogbein et al., 2024; Park et al., 2020). However, despite several papers describing BTZ-induced apoptosis in different tumor cell lines (Zheng et al., 2015; Swiderska-Kolacz et al., 2025; Liu et al., 2023), its clinical efficacy in solid tumors has been compromised by side effects including renal toxicity and neuropathy, which restrict its unconditional use (Sogbein et al., 2024). This paper provides evidence that BTZ is efficacious in reducing the viability of HCT116 and HT29 colon cancer cells at a concentration range up to 15 nM, which is considerably lower compared to the corresponding plasma concentrations obtained in MM patients (Sogbein et al., 2024). To date, poor clinical data exist on the use of bortezomib for colon cancer treatment (Feng et al., 2022; Mao et al., 2024), probably because of the limitations in clinical applications of this drug in solid tumors. Nevertheless, some pre-clinical evidence encourages the association of BTZ with other antitumor agents in colon cancer models to get synergistic effects (Caldiran et al., 2023; Celesia et al., 2022b; Park et al., 2018; Qing et al., 2021). Indeed, the combination approach is one of the most promising tools to reduce the drug concentrations and limit side effects when translated *in vivo*. This paper represents the first evidence that a HDAC6 inhibitor exerts a synergistic apoptotic effect with BTZ in colon cancer cells. Specifically, we referred to ITF3756, a selective HDAC6 inhibitor produced by Italfarmaco. This compound has been previously tested in breast, leukaemia and melanoma cells (Zamperla et al., 2024), and this manuscript represents the first study describing its efficacy in colon cancer models. HDAC6 is a unique HDAC member that deacetylates cellular proteins regulating the cell fate (Zheng et al., 2023) and has recently emerged as an intriguing antitumor target (Oliveira-Silva et al., 2025b).

Considering the involvement of HDAC6 in lipid metabolism, the original idea was to block HDAC6 to clarify the effects on lipogenesis in colon cancer cells. To this purpose, the study was conducted in two colon cancer cell lines, HCT116 and HT29, that differ in HDAC6 expression levels (Zhang et al., 2019) and may also display different lipid metabolism profiles. Our results indicate that HT29 contain a higher basal level of lipid droplets compared to HCT116 cells and were less responsive to the HDAC6 inhibitor either alone or in combination with BTZ. The two compounds, ITF3756 and BTZ, were able to increase lipogenesis either when used alone at active concentrations or in combination at subtoxic doses.

Given the higher susceptibility of HCT116 cells, the attention was focused on this cell line to characterise the cell death mechanism. Combination of the two compounds at sub-toxic doses in these cells produced synergistic interaction and apoptosis as evidenced by annexin V positivity and activation of apoptotic markers. It is interesting to note that apoptosis was accompanied with TP53 increase, a well-known tumor suppressor and proapoptotic factor, which is wild type in HCT116 and mutated in HT29 cells, a condition that may further explain the different response of the two cell lines.

Moreover, quantification of triacylglycerols content and activation of lipogenesis markers including phospho-mTOR, SREBP-1and PPARγ confirmed in HCT116 cells the pro-lipogenic action of the ITF3756/BTZ combination. SREBP-1 is well known to exert a crucial role in lipogenesis. Specifically, in the mature, cleaved form SREBP-1 acts as a transcription factor regulating genes related to cholesterol biosynthesis, fatty acid synthesis, and lipid production. The nuclear localization of cleaved SREBP-1 in HCT116 cells confirmed its involvement in ITF3756/BTZ-mediated lipogenesis. Lipogenesis is known to exert a double role in tumor cells. On the one hand, it represents an important metabolic hallmark of cancer, which has been associated with tumor cell survival and progression (Mounier et al., 2014). On the other hand, increased lipid accumulation can promote lipophagy, the process of autophagic degradation of lipid droplets and a form of cell death that can be exploited to target tumor cells (Xu et al., 2016; Mukhopadhyay et al., 2017). The results presented in this paper indicate that lipogenesis induced by the ITF3756/BTZ combination exerts a pro-survival function. Indeed, either lipogenesis inhibitors, Diacylglycerol-O-acyltransferase inhibitors (DGATi) or SREBP-1 silencing increased the antitumor efficacy of the combination. DGAT-1 and DGAT-2 are involved in lipid metabolism homeostasis since they exert a crucial role in lipid droplet formation (Wang et al., 2024; Deng et al., 2024; Cheng et al., 2020). These enzymes can largely compensate each other for triacylglycerol storage (Jin et al., 2014). Therefore, inhibition of both enzymes concomitantly using A922500 (DGAT-1 inhibitor) and PF-06424439 (DGAT-2 inhibitor) guaranteed complete blockage of lipid droplet formation as clearly evidenced by ORO staining. In this condition, the effects of the ITF3756/BTZ combination on cell viability and morphology were significantly exacerbated. Interestingly, these effects were observed in both HCT116 and HT29 cells, thereby reinforcing the hypothesis of the pro survival role of lipogenesis. In addition, SREBP-1 silencing in HCT116 cells augmented the effects of the combination, thus confirming that SREBP1-mediated lipogenesis exerts a pro-survival action. These data are in accordance with the findings of Wang et al., who found that SREBP-1 silencing inhibits the proliferation of human esophageal squamous carcinoma cells (Wang et al., 2020). The pro-tumoral role of SREBP-1 has been described in other tumor types, including renal cell carcinoma (Shen et al., 2021) and non-small cell lung cancer (Tiong et al., 2022). He et al. have recently provided a review describing the mechanisms and implications of SREBP-1 in cancer progression and chemoresistance (He et al., 2024). Similarly, Zhao et al. have focused on SREBP-1 targeting as a strategy for cancer (Zhao et al., 2022).

Overall, this paper suggests that the ITF3756/BTZ combination is a promising tool for potential colon cancer treatment. Moreover, inhibiting lipogenesis may represent a favourable condition that improves the synergistic action of the two drugs. Obviously, the clinical feasibility of combining DGAT inhibitors/SREBP-1-targeted drugs with ITF3756/BTZ is beyond the scopes of this manuscript, which represents a preliminary *in vitro* evaluation. Encouraging data sustain the clinical use of either selective HDAC6 inhibitors (Amengual et al., 2021) or DGAT inhibitors (Deng et al., 2024) as anti-tumor agents. According to our observations, pre-treatment with DGAT1 and DGAT2 inhibitors together guarantees complete blockage of lipogenesis, suggesting a hypothetical sequence of administration. However, clinical evaluations and toxicity profiles remain to be determined.

## Data Availability

The original contributions presented in the study are included in the article/Supplementary Material, further inquiries can be directed to the corresponding author.
