# Supplementary figures and images for: Targeting lipogenesis promotes the synergistic effect of the selective HDAC6 inhibitor ITF3756 with bortezomib in colon cancer cells
Source: Front Pharmacol. 2025 Dec 12;16:1706770. doi: 10.3389/fphar.2025.1706770 (PMC12742218; doi:10.3389/fphar.2025.1706770)

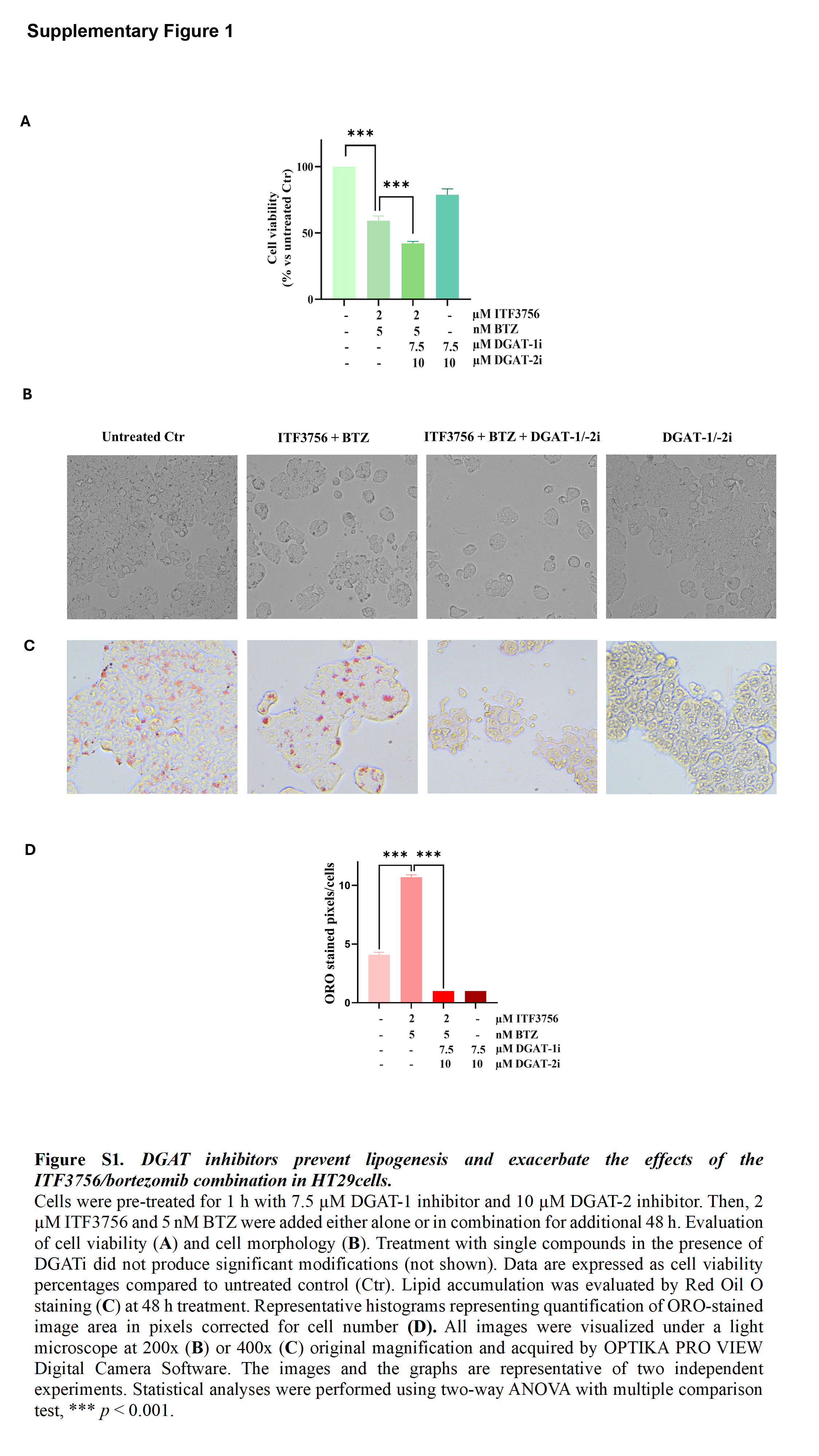

Supplement: Supplementary file 1 [file Image1.tif]
